# Diagnosing and management of thyroid nodules and goiter – current perspectives

**DOI:** 10.1007/s12020-024-04015-8

**Published:** 2024-08-31

**Authors:** Katica Bajuk Studen, Bartosz Domagała, Simona Gaberšček, Katja Zaletel, Alicja Hubalewska-Dydejczyk

**Affiliations:** 1 Department of Internal Medicine, Faculty of Medicine, University of Ljubljana, Ljubljana, Slovenia; 2 Division of Nuclear Medicine, University Medical Centre, Ljubljana, Slovenia; 3 Department of Endocrinology, Oncological Endocrinology, Nuclear Medicine and Internal Medicine, University Hospital, Krakow, Poland; 4 Chair and Department of Endocrinology, Jagiellonian University Medical College, Krakow, Poland

**Keywords:** Thyroid nodules, Minimally invasive techniques, Laser ablation, Thyroid ultrasound

## Abstract

Due to the frequent diagnosis of benign thyroid nodules, it is necessary to deviate from the traditional paradigm based on frequent surgical treatment. This article highlights the evolution of diagnosis and treatment in recent years, beginning from standardization of ultrasound assessment of nodules and cytology results to minimally invasive techniques to reduce the size of symptomatic thyroid nodules. These achievements reduce the number of surgeries, enable more individualized care for patients with benign thyroid disease, reduce long-term complications, and promote cost-effectiveness within healthcare systems. Furthermore, although the use of minimally invasive techniques significantly decreases thyroid nodule volume, the thyroid nodule usually does not disappear and the challenges in this field are discussed (the efficacy of thermal ablation, a variable part of thyroid nodules that remains viable after thermal ablation, some of the nodules treated with thermal ablation may require a second treatment over time and the efficacy of thermal ablation in nodules with different phenotypes). However, although surgery still represents the “gold standard” for establishing the final histopathologic diagnosis, it is associated with lifelong thyroid hormone substitution need and serious complications in rare cases. Therefore, it should represent the *ultima ratio* only after a detailed diagnostic procedure. In the future, artificial intelligence-assisted programs for the evaluation and management of nodules are expected.

## Introduction

Thyroid nodules are very common in the adult population, and most of them are benign [1, 2]. Although salt iodinization has led to a decreased incidence of diffuse goiter, the incidence of thyroid nodules has increased [3]. This increase is likely due to the widespread use of diagnostic imaging, increased medical surveillance, and improved access to health care in recent decades [3, 4]. As a result, a significant number of thyroid nodules emerge and require attention [5]. Up to 60% of adults in the general population have one or more thyroid nodules [1]. The main challenge in the current management of thyroid nodules is avoiding overdiagnosing low-risk cancers without overlooking advanced or higher-risk tumors that require immediate treatment as overdiagnosis leads to overmedication and overtreatment [4]. The treatment of benign thyroid nodules has also diversified in recent decades, offering more options for patient-tailored treatment than there were available in the past. The aim of this article is to review new approaches for the diagnosis and treatment of thyroid nodules. In this review, management of thyroid cancer is not included.

## Diagnosing thyroid nodules

Significant advances in the diagnostic evaluation of thyroid nodules have been made in recent decades, including improvements in the quality of ultrasound examination. Ultrasound features that have been consistently associated with malignancy include hypechogenicity, infiltrating, irregular or lobulated margins, intranodular microcalcifications, and a taller- than- wide shape [4]. However, the diagnostic sensitivity and specificity of these features vary, and a considerable inter-observer variability has been reported [6]. In a blinded evaluation of video recordings of the ultrasound examinations of 123 thyroid nodules by 7 experts, the Gwet’s AC1 inter-rater coefficients (on a scale from 0.0 to 1.0) were 0.34, 0.53, 0.72, and 0.79 for the four most important features in decision making, i.e. irregular margins, microcalcifiactions, echogenicity, and extrathyroidal extension, respectively. Extrathyroidal extension was correctly identified in just 45.8% of the cases [7]. Therefore, several professional organizations such as the European Thyroid Association (ETA), the American Thyroid Association (ATA), the American Association of Clinical Endocrinologists/American College of Endocrinology/Assoziazone Medici Endocrinologi, and the Korean Society of Thyroid Radiology, among others, have developed several sonographic stratification systems (e.g., Thyroid Imaging Reporting and Data Systems, TIRADS) for reporting malignancy risk on ultrasound examination [7, 8]. Currently, 20 risk stratification systems for thyroid nodules are in use [6]. Several studies have reported varying diagnostic efficacy of these systems [9, 10]. In a study comparing KWAK-TIRADS, American College of Radiology (ACR) TI-RADS and 2015 ATA guidelines, KWAK-TIRADS and ATA guidelines showed a better diagnostic efficiency than ACR TI-RADS [10]. ACR TI-RADS demonstrated a higher specificity (79.7%) whereas ATA US pattern had a higher sensitivity (95.5%). All three systems performed better in differentiating nodules >1 cm. In Poland, a modified EU-TIRADS classification (EU-TIRADS-PL) was introduced in 2022. The most important change is the earlier indication for biopsy in the most suspicious lesions (EU-TIRADS-PL 5) compared to the EU-TIRADS scale (≥5 mm vs. >10 mm, respectively) [11]. Furthermore, due to some degree of heterogeneity across systems regarding the definition of certain nodule features, an international expert consensus on an ultrasound lexicon for thyroid nodules has recently been published (I-TIRADS), with the aim of creating an international system for malignancy risk stratification of thyroid nodules in the future [6]. New approaches to malignancy risk assessment at ultrasonography include the use of artificial intelligence based software applications for computer-aided diagnosis. Lai et al. developed several AI models (traditional feature-based machine learning models, deep convolutional neural networks and a fusion model) and showed that their fusion diagnostic model outperformed the experienced radiologist in diagnosing TI-RADS 4 nodules with the area under the receiver operating curve of 0.880 [12]. In a study by Chen et al., the area under the curve and sensitivity of an AI model based on TI-RADS exceeded those of junior radiologists; the specificity of the model was higher than that of both experienced and junior radiologists [13]. However, due to the widely demonstrated overdiagnosis of benign and malignant thyroid lesions without a significant change in thyroid cancer mortality, ultrasound screening of asymptomatic adults is discouraged [14].

Ultrasound-guided fine needle aspiration biopsy remains the main method for ruling out malignancy in thyroid nodules, with reports relying on the Bethesda System for Reporting Thyroid Cytopathology [15]. The third edition, published in 2023, provides several updates, the most important being the assignment of a single name to each of the 6 diagnostic categories: (I) nondiagnostic; (II) benign; (III) atypia of undetermined significance; (IV) follicular neoplasm; (V) suspicious for malignancy; and (VI) malignant [14]. Each category has an implied malignancy risk that has been updated and refined based on data reported after the second edition (summarized in Table 1).

**Table 1:** Potential risk of malignancy (depending on the fine-needle aspiration category) according to the Bethesda 2023 system [adapted from ref. [15]]

FNA category	Terminology according to the 2023 Bethesda System	Mean risk of malignancy (%, [range])
I	Nondiagnostic	13 [5–20]
II	Benign	4 [2–7]
III	Atypia of undetermined significance (with nuclear or non-nuclear atypia)	22 [13–30]
IV	Follicular neoplasm	30 [23–34]
V	Suspicious for malignancy	74 [67–83]
VI	Malignant	97 [97–100]

The cytology result plays a key role in optimizing subsequent management. However, there are several diagnostic pitfalls of the Bethesda system that can lead to false-positive, false-negative, non-diagnostic or indeterminate results [16]. In addition, core needle biopsy can provide complementary information for clinical management in anaplastic thyroid tumors, lymphomas and metastatic lymph nodes [17]. However, its use to differentiate benign nodules from follicular neoplasms in lesions with indeterminate cytology is not supported by adequate evidence [17, 18]. Molecular testing can improve diagnostic outcomes in thyroid nodules with indeterminate cytology since the histological diagnosis of these nodules includes benign tumors and malignant neoplasms, including folicullar and papillary thyroid cancer [19]. The principal use of molecular markers in this case is diagnostic, i.e. »ruling-out« or »ruling-in« thyroid cancer, with implications for further management [20]. In 2014, the Cancer Genome Atlas Research Network studied molecular profile of 496 papillary thyroid cancers, mainly classicial and folllicular variants, and delivered two distinct molecular profiles: BRAF^V600E^-like and RAS-like [21]. Indeterminate nodules have a malignancy risk ranging from 20 to 30% and less than 5% of these are positive for BRAF^V600E^ [22]. The remaining part of indeterminate nodules, negative for this mutation, are mostly benign nodules, follicular adenomas, or tumors with follicular pattern, thus having RAS-mutational profile [19]. The presence of RAS-like mutations confirms the presence of a clonal process but cannot discriminate between benign, malignant, or low-risk lesions, such as non-invasive follicular thyroid neoplasms with papillary-like nuclear features (NIFTP) [23]. The currently available molecular tests for indeterminate nodules range from small panels such as the 7-gene panel to panels containing a high number of genes. Their use is increasing and their continuous improvement will likely implement them as part of routine indeterminate thyroid nodule evaluation [19].

In addition, autonomously functioning thyroid nodules often have multiple ultrasound features associated with malignancy and may lead to indeterminate cytology results [24]. This problem can be alleviated by performing a [99mTc]Tc-pertechnetate thyroid scan prior to biopsy [25, 26]. This issue was recently addressed in the European Association of Nuclear Medicine (EANM) response to the 2023 ETA clinical practice guidelines for the management of thyroid nodules, as for example in Germany, up to 20% of all thyroid nodules are autonomus, with 80% being found in individuals with normal TSH levels [26]. By contrast, in Slovenia, an area with previous mild iodine deficiency, the proportion of euthyroid patients with thyroid autonomy increased from 14.4% to 32.4% after improvement in iodine intake [27]. Furthermore, [99mTc]Tc-MIBI or [18F]FDG-PET/CT scans can safely prevent unnecessary diagnostic surgery in about half of patients with cytologically indeterminate nodules [20]. It is expected that artificial intelligence (AI) and machine learning systems will improve and streamline the diagnostic process in the future [20].

## Management of thyroid nodules

**Fig. 1:**
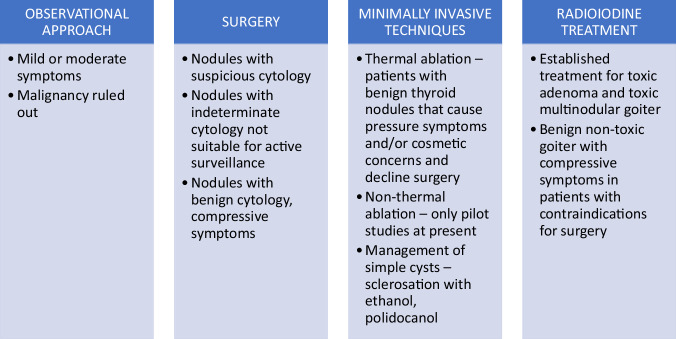
Current options for management of patients with thyroid nodules

Currently, there is no worldwide consensus on the management of patients with non-toxic goiter, as there is no ideal treatment option [5, 28, 29]. Since most thyroid nodules are benign, asymptomatic and do not require treatment [5], an observational approach is preferred in patients with mild or moderate symptoms, and in whom malignancy has been ruled out [4]; and was demonstrated to be safe [30] (Fig. 1). Regarding medical therapy of goiter, many clinicians would still prescribe levothyroxine for euthyroid patients with simple goiter although there is no such recommendation in current guidelines [5, 31]. Specifically, there is no agreement regarding its efficacy, and several authors have demonstrated that sufficient goiter reduction can be established only in a limited number of patients [32–35]. Notably, for obtaining goiter reduction, levothyroxine therapy aiming at suppressing serum TSH concentrations is often prescribed leading to subclinical or even overt hyperthyroidism [32]. This situation should be avoided, particularly in elderly patients due to untoward side effects on the cardivascular and skeleton systems [36, 37]. Although goiter development is strongly associated with iodine deficiency, iodine supplementation is also not recommended for thyroid nodule management by current guidelines since there is limited information about its efficacy in patients with already established nodular goiter and a sudden increase in iodine intake may induce thyrotoxicosis in predisposed individuals [5, 32].

Currently, surgery is only one of the possible treatment options for patients with nodular thyroid disease, as sensitive diagnostic tools such as ultrasonography and ultrasound-guided fine-needle aspiration biopsy have been developed in recent decades. These advances allow active surveillance and the increasing use of minimally invasive techniques (MIT) for nodule ablation [5, 38]. As not all reasons for surgery are reported in the available literature, some patients with thyroid nodules undergo surgery solely for diagnostic purposes, resulting in an unfavorable risk-benefit and cost-benefit ratio [5, 39, 40]. There is a significant need for more cost-effective, risk-adapted approaches to the treatment of this condition, taking into account the clinical context, medical expertise, available technology and patient preferences [5].

### Surgery

In the past, patients with non-toxic benign thyroid nodules causing pressure symptoms were offered surgical treatment. Currently, surgery is only one of the possible treatment options for patients with nodular thyroid disease; however, it represents the “gold standard” for definitive histopathologic diagnosis [5, 38]. According to the 2023 ETA Clinical Practice Guidelines for thyroid nodule management, surgery may be appropriate in several scenarios: for symptomatic thyroid nodules, as an alternative to MIT and radioiodine therapy (RAI), for nodules that have been citologically classified as benign and/or classified as low-risk on ultrasound (US) and become symptomatic over time, for nodules with indeterminate cytology that are unsuitable for active surveillance, and for nodules with suspicious cytology [5]. Notably, by far the most important indication for thyroid surgery is the elimination of a malignant nodule. A recently published large retrospective study including 17,592 patients diagnosed with a thyroid nodule >1 cm which were followed up to 23 years reported a malignancy rate of only 1.1% which is much lower than previously reported [41]. In most patients with multiple nodules involving both lobes of the gland, total thyroidectomy is often required, and consenquently, the patient needs lifelong levothyroxine supplementation [4]. There is no consensus on the procedure of choice for patients with asymmetric nodular goiter [4]. In selected cases, lobectomy may be considered a safer alternative to total thyroidectomy. However, it requires long-term follow-up due to the risk of nodule recurrence and potential need for a second operation [4]. Total thyroidectomy is associated with a signifcantly higher risk of complications compared to lobectomy even among high-volume surgeons [39]. In a German study aiming to evaluate short-term complications of goiter surgery that included 12,888 patients undergoing thyroid surgery in a 15-month period, the rates of unilateral and bilateral transient nerve palsy were 3.6% and 0.07% of nerves at risk, the rate of transitory hypoparathyroidism was 15.3%, the rate of postoperative bleeding was 1.4% and the rate of postoperative infection was 0.07%, respectively [40]. In the last decades, the benefits of neuromonitoring during thyroid surgery which enables both the confirmation of anatomical integrity and the assessment of laryngeal nerve function have determined its widespread acceptance [42].

### Minimally invasive techniques

#### Thermal ablation techniques

In 2000, after a series of tests on experimental models, a feasibility study was published showing that ultrasound-guided thermal ablation (TA) with a laser source can be performed on thyroid lesions easily and with minimal discomfort and risk to the patient [43]. In the following years, ultrasound-guided ablation techniques using different energy sources were proposed, some of which are currently routinely used in clinical practice [44]. Compared to thyroidectomy, these techniques are minimally invasive, can be performed on an outpatient basis, have a lower complication profile and offer greater potential for preserving thyroid hormone function, and are also less expensive. Clinical results have shown that these techniques are similarly effective and safe. Laser ablation (LA) was the first TA technique to be introduced into clinical practice for the treatment of nodules [45], followed by radiofrequency ablation (RFA) [46]. Experience with microwave and high-focused ultrasound ablation is still much more limited [47].

In LA of thyroid nodules, 1–4 optical fibers are introduced at a distance of 10 mm along the long axis of the nodule or via a transisthmic approach using thin and flexible 21-gauge needles [44]. The heat-induced changes in the tissue are visualized by the appearance of hyperechoic signals caused by the formation of gas microbubbles. During the procedure, the glass fibers are moved backwards (“pull-back”), which can destroy large parts of the thyroid nodule [48].

In RFA of thyroid nodules, an electric field generated by a radiofrequency generator connected to an internally cooled electrode needle creates a frictional motion at the ionic level, which leads to heat generation. The electrode is inserted into the target nodule under ultrasound guidance, and continuous repositioning of the applicator (“moving shot” or “multiple overlapping shot” technique) results in an ellipsoidal necrotic area [49].

Microwave ablation uses an electromagnetic field to induce oscillation of polarized particles, converting the kinetic energy into heat when the particles collide. An internally cooled shaft antenna is inserted into the target lesion under ultrasound guidance. The nodule is divided into smaller conceptual ablation units and treated sequentially, with each unit being exposed to microwave energy for 5–10 s until the entire nodule appears hyperechoic on the ultrasound image [50].

High-intensity focused ultrasound is a non-invasive technique that delivers energy to a small area by focusing high-intensity sound waves from numerous sources onto a specific target area. The energy conversion causes tissue vibration and frictional heat that generates high temperatures of over 85 °C. This process leads to vaporization of the tissue, expansion and collapse of the microbubbles and cell death. The tissue outside the focused zone receives only a low density of acoustic energy and remains unharmed [50, 51].

Although the use of TA significantly reduces the volume of the thyroid nodule, the thyroid nodule usually does not disappear completely [46]. Therefore, follow-up studies usually distinguish between technique efficacy (i.e. volume reduction ratio, VRR, ≥50% at least 6 months after treatment) and nodule regrowth (≥50% increase compared to the smallest previous volume on US examination) [46, 52]. With TA, a mean VRR of 50–90% can be achieved at 12 months, and this result is maintained for up to 5 years [45, 47]. Such a result can be achieved in the majority of patients, but the extent of nodule reduction can vary greatly and cannot be accurately predicted [47]. After a single TA treatment, about one third of nodules treated with LA and about 20% of nodules treated with RFA have a VRR of less than 50% at 12 months [45, 47, 53]. It has also been reported that the lower the 12-month VRR, the higher the risk of recurrence and the shorter the time to regrowth [54]. A variable portion of thyroid nodules remains viable after TA, and up to 15% of nodules treated with TA may require a second treatment over time. In a recent study reporting the results of repeat TA treatment (LA and RFA) in nodules that had decreased in volume by less than 50% after the first procedure (suggesting ineffectiveness of the technique), retreatment resulted in VRR of 50% and 52.2% at 6 and 12 months, respectively [46]. Inadequate treatment at the edge of the nodule is one of the possible factors contributing to nodule regeneration [55]. When comparing the efficacy of LA and RFA ablation, a recent meta-analysis found that LA achieved the highest VRR at 6 months, while RFA showed better results at 1 and 12 months [56]. One possible explanation for this difference is that with RFA, the electrode tip is moved gradually over the entire nodule region. This approach allows for more individualized therapy and tailored treatments based on the specific characteristrics of the nodules, maximizing the ablation of the lesion at the margin [57].

In another study, it was reported that 28% of patients experienced nodule regrowth after TA (LA and RFA), with recurrence of clinical symptoms in 26% of cases where regrowth occurred and a second treatment was required in 28% of those cases [45]. This study also reported that 11% of patients underwent surgery during follow-up. Non-benign histopathology was found in 3.9% of all treated patients (2.4% if microcarcinomas were excluded), including follicular carcinomas, papillary carcinomas, and follicular tumors of uncertain malignant potential. There was no difference in the rate of non-benign pathology between patients who had undergone one or two fine needle aspiration biopsies previously. On the basis of these results, the authors of the study suggested that in patients with a VRR of less than 20% after the initial TA procedure, a repeat cytologic evaluation, and possibly surgery may be more appropriate than a repeat TA procedure.

Furthermore, TA exibits varying efficacies in nodules with different phenotypes [47]. A retrospective 4-year follow-up study showed that LA is more effective for spongiform nodules than for solid nodules [58]. The size of spongiform nodules progressively decreased over 48 months. The study also reported that spongiform nodules with intra-nodular vascularity shrank more than nonspongiform nodules with peripheral vascularity, probably due to better heat distribution in the former [59]. It is noteworthy that to shrink nodules with a median initial volume of 11.59 ml by about 60%, a median energy of 5004.9 J should be administered (the strength of one fiber is 6000 J according to the manufacturer’s instructions) [unpublished data]. This effect is mainly achieved in lesions with a predominantly liquid component. However, for almost completely solid lesions, the energy required to achieve a similar effect can be as much as three times higher and the treatment needs to be repeated, making it less cost-effective. Further research should define more precisely the clinical and ultrasound characteristics of nodules that respond best or inadequately to TA treatment. In addition to ultrasound, future studies may explore the use of molecular markers associated with cell proliferation, spectral analysis to categorize nodule composition, or artificial intelligence to predict TA efficacy [47, 60].

The ocurrence of minor and major complications of TA is reported to be low and is strongly related to the operator’s expertise [44, 61]. In a large multicenter study, including 1,531 patients undergoing LA, a 0.5% rate of major complications was reported, with temporary vocal cord palsy documented in 8 patients [62]. However, in 6 of the 8 patients, the target nodule was located in a “danger area” close to the infero-medial portion of the thyroid lobe. Therefore, this paratracheal thyroid zone should be carefully avoided in benign thyroid lesions. Minor complications (sub-capsular and peri-thyroidal hematomas and skin burns) were reported in 0.5% of patients. A meta-analysis analyzing the safety of RFA and including 2421 patients reported a 2.38% rate of overall and a 1.35% rate of major complications [63]. Among minor complications, hematomas, skin burns, persistent pain, vaso-vagal reactions, and vomiting were reported.

However, confidence in the long-term treatment outcomes of TA for benign thyroid nodules compared to surgery is still needed. Indeed, only a few studies have examined the efficacy of TA techniques beyond 5 years. In a recently published meta-analysis, 5-year follow-up reports on TA for benign thyroid nodules were analyzed, including five studies with a total of 939 patients [64]. This meta-analysis showed a mean final VRR of 74.48%. Additionally, 9.6% of patients underwent a second procedure, and 10.6% of patients were found to have a regrowth of the nodule. Moreover, a 2020 ETA survey reported that TA procedures for benign thyroid nodules were available to, on average, only 16% of respondents and performed by only about 5% of respondents. In contrast, thyroid ultrasound, fine needle aspiration biopsy and thyroid surgery were available in virtually every hospital or clinic that participated in the survey [18]. Among the possible reasons for this limited availability, the absence of specialized training courses and the lack of adequate and consistent reimbursement policies by the national health services were proposed. Since then, guidelines for TA procedures for benign thyroid nodules have been published by several professional organizations [5, 48, 65–67].

#### Non-thermal ablation techniques

TA techniques are still limited by the risk of thermal spread and injury to adjacent structures. Therefore, there is growing interest in exploring the role of non-thermal ablation techniques, particularly cryoablation and irreversible electroporation [50]. Cryoablation uses circulating cooled liquids such as nitrogen or argon, which rapidly expand into gas and generate temperatures as low as –190 °C. Ice crystals form, physically damaging cell membranes and disrupting metabolic functions, leading to cell necrosis [68]. A pilot study using cryoablation in 3 patients with benign thyroid nodules was recently published [69]. In irreversible electroporation, irreversible permeabilization of the cell membrane is achieved by microsecond-long, high electric field pulses [50]. A case report of a patient with recurrent thyroid cancer has been described [70]. Currently, more robust studies are needed to evaluate the role of these techniques.

## Management of simple cysts

According to the 2023 ETA Clinical Practice Guidelines for thyroid nodule management, ethanol ablation is preferred as an effective, safe and cost-effective treatment for cystic or predominantly cystic symptomatic thyroid nodules [5]. However, some patients may experience burning pain at the injection site when ethanol penetrates the subcutaneous tissue, or more serious side effects such as vocal cord paresis or extraglandular fibrosis [54–56]. A recently published meta-analysis reported the overall prevalence of complications of percutaneous ethanol injection was 32%; with the prevalence of minor complications of 32% and the prevalence of major complications of 2% [71]. The pooled prevalence rate of local pain was 21% and the pooled prevalence rate of dysphonia was 1%. Compared to medical ethanol, polidocanol is more accessible and has a more moderate sclerosing effect. Sclerotherapy with polidocanol is an alternative to ethanol for the treatment of benign cystic and predominantly cystic thyroid nodules [57]. It is also a safer agent for use without reaspiration and can be left in the cyst cavity to achieve complete adhesion with only one application [57]. Furthermore, percutaneous polidocanol sclerotherapy has been reported to reduce the volume of large, partially cystic thyroid nodules, thereby facilitating the subsequent TA (RFA) procedure [58].

## Radioiodine treatment

RAI therapy is an established treatment for toxic adenomas and toxic multinodular goiter [29]. In addition, according to the recently published EANM guideline on RAI therapy for benign thyroid disease, RAI therapy is indicated in patients with benign, non-toxic goiter with compressive symptoms and a total thyroid volume of less than 100 ml, especially in elderly patients, in patients with significant comorbidities (e.g. cardiovascular), or in patients with a history of neck surgery [29]. In addition, RAI treatment is indicated for the treatment of large non-toxic goiter with intrathoracic extension when surgery is not feasible or is refused by the patient. A pilot study reported that the combination of RAI and LA in large hyperfunctioning nodules causing compression symptoms resulted in a more rapid reduction of nodule volume and faster improvement of local symptoms than RAI therapy alone [72].

## Conclusion

Although thyroid nodules and benign nodular goiter are common findings, very few of them warrant treatment after the initial diagnostic evaluation. Recent technological advances, including improvements in ultrasound equipment and software and the development of MIT ablation techniques, have significantly reduced the number of patients undergoing surgery. Studies are still needed to improve the nodule selection and optimize the standard technique for MIT to minimize clinical failures. In the future, AI-assisted programs for nodule evaluation and management are expected to play a significant role.
